# Spaceflight influences gene expression, photoreceptor integrity, and oxidative stress-related damage in the murine retina

**DOI:** 10.1038/s41598-019-49453-x

**Published:** 2019-09-16

**Authors:** Eliah G. Overbey, Willian Abraham da Silveira, Seta Stanbouly, Nina C. Nishiyama, Gina D. Roque-Torres, Michael J. Pecaut, David Carl Zawieja, Charles Wang, Jeffrey S. Willey, Michael D. Delp, Gary Hardiman, Xiao Wen Mao

**Affiliations:** 1 University of Washington, Department of Genome Sciences, Seattle, WA USA; 2 Queen’s University Belfast, Faculty of Medicine, Health and Life Sciences, School of Biological Sciences, Institute for Global Food Security (IGFS), 19 Chlorine Gardens, Belfast, Northern Ireland BT9 5DL UK; 3 Department of Basic Sciences, Division of Biomedical Engineering Sciences (BMES), Loma Linda University, Loma Linda, CA 92350 USA; 4 Center for Dental Research, Loma Linda University, CA, 92354 USA; 5 Department of Medical Physiology, Texas A&M University, College Station, Texas USA; 6 Center for Genomics, School of Medicine, Loma Linda University, Loma Linda, CA 92350 USA; 7 Department of Radiation Oncology, Wake Forest School of Medicine, Winston-Salem, NC 27157 USA; 8 Department of Nutrition, Food and Exercise Sciences, Florida State University, Tallahassee, FL 32306 USA

**Keywords:** Transcriptomics, Transcriptomics, RNA sequencing, Gene expression, Experimental models of disease

## Abstract

Extended spaceflight has been shown to adversely affect astronaut visual acuity. The purpose of this study was to determine whether spaceflight alters gene expression profiles and induces oxidative damage in the retina. Ten week old adult C57BL/6 male mice were flown aboard the ISS for 35 days and returned to Earth alive. Ground control mice were maintained on Earth under identical environmental conditions. Within 38 (+/−4) hours after splashdown, mice ocular tissues were collected for analysis. RNA sequencing detected 600 differentially expressed genes (DEGs) in murine spaceflight retinas, which were enriched for genes related to visual perception, the phototransduction pathway, and numerous retina and photoreceptor phenotype categories. Twelve DEGs were associated with retinitis pigmentosa, characterized by dystrophy of the photoreceptor layer rods and cones. Differentially expressed transcription factors indicated changes in chromatin structure, offering clues to the observed phenotypic changes. Immunofluorescence assays showed degradation of cone photoreceptors and increased retinal oxidative stress. Total retinal, retinal pigment epithelium, and choroid layer thickness were significantly lower after spaceflight. These results indicate that retinal performance may decrease over extended periods of spaceflight and cause visual impairment.

## Introduction

We are entering an era of renewed interest in space exploration. Although officially the space race ended decades ago, humanity is crossing the threshold into a second space age that includes new global partnerships and private companies. This new infrastructure and organization has brought the concept of long duration space missions, such as permanent moon settlements and manned missions to Mars, from a distant goal to a tangible reality^1^. However, the space environment is fundamentally different from Earth. Microgravity and ionizing radiation compose a unique set of physiological stressors, the effects of which remain to be fully characterized^2,3^.

In humans, one of the most apparent physiological responses to the space environment is the redistribution of fluid throughout the body that occurs due to microgravity. This redistribution shifts fluid upward from the lower parts of the body^4^. An increased intracranial pressure caused by this fluid shift has been attributed as a major cause of spaceflight-associated neuro-ocular syndrome (SANS)^5^. 40% of astronauts have experienced SANS^6^, including the spaceflight subject of the NASA twins study^7^. The physiological impact of SANS encapsulates multiple components of the eye and includes optic disc edema, globe flattening (GF), choroidal and retinal folds, hyperopic refractive error shifts, and nerve fiber layer infarcts (i.e., cotton wool spots)^5,8^. While the physiological characteristics of SANS are well documented, the mechanisms that drive SANS are still poorly understood.

To date, despite evidence showing ocular functioning impairment following exposure to the space environment, few studies have investigated molecular mechanisms involved in the spaceflight-associated changes in retinal structure and function. A previous study from our group characterized the effects of a 13-day exposure to the space environment aboard the Space Shuttle Atlantis (STS-135) in mice. This study found that spaceflight conditions induced oxidative damage that resulted in mitochondrial apoptosis in the mouse retina^2^. Subsequent work with low-dose proton radiation and a simulated microgravity mice model showed a major role of simulated space radiation in the increase of oxidative stress, with synergistic effects when simulated microgravity was applied^9,10^. In our current study, mice were exposed to the space environment for 35 days aboard the International Space Station (ISS) to determine whether the space environment induces oxidative damage on ocular structure and to characterize gene expression profiles of mouse retina exposed to spaceflight.

## Results

### The space environment causes changes in gene expression related to visual and RNA regulation pathways

**Figure 1:**
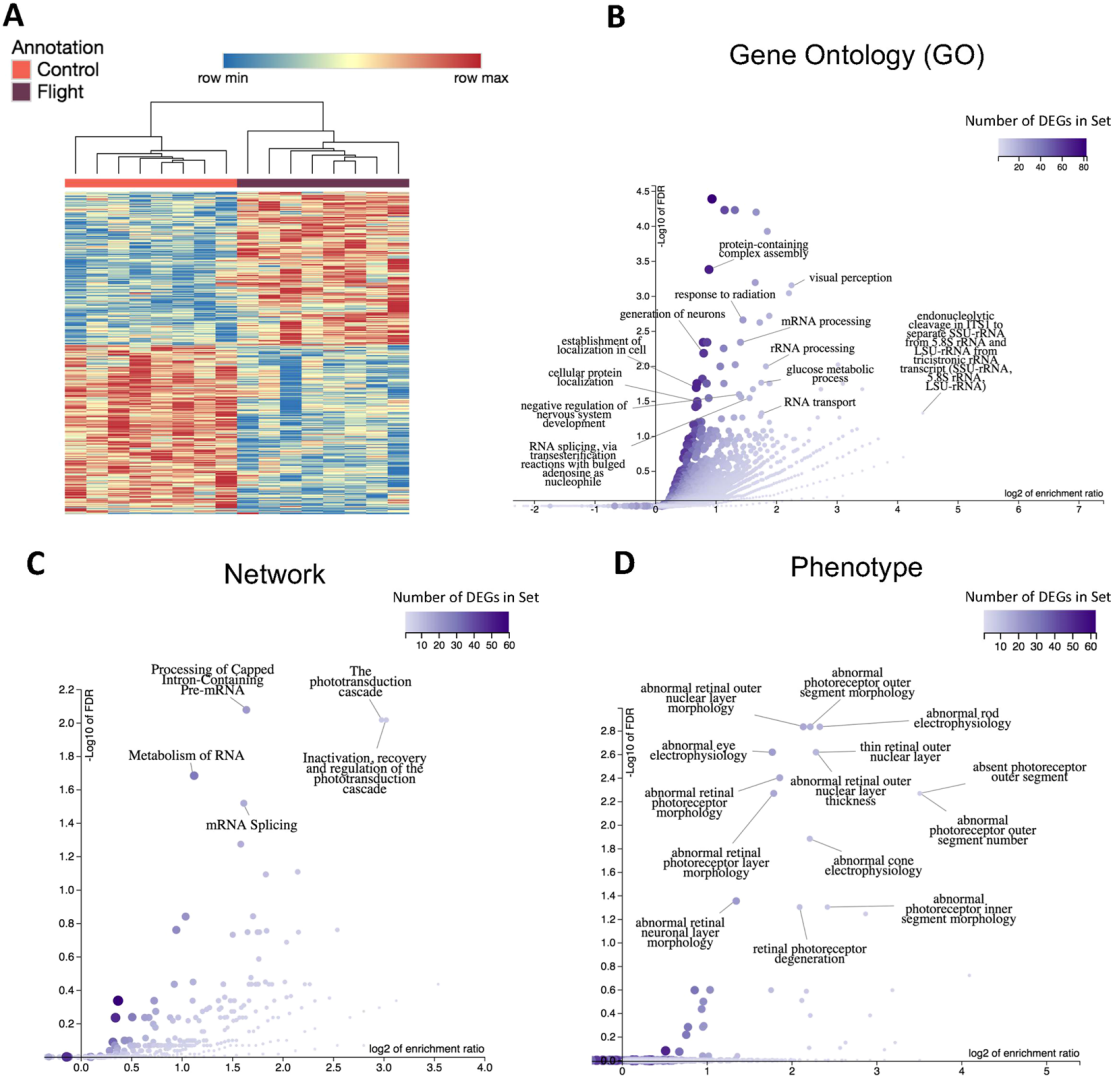
DEG clustering and functions between spaceflight and control mice. (**A**) Hierarchical clustering of the 600 DEGs between spaceflight and control mice using an adjusted p-value threshold of 0.1. The spaceflight group had 286 upregulated genes and 314 downregulated genes compared to the ground control; **(B)** Enriched gene ontology (GO) biological process categories for DEGs. The affinity propagation option from WebGestalt was applied to the select representative display categories; **(C)** Enriched networks among DEGs from the Reactome database; **(D)** Enriched phenotypes impacted by the DEGs from the Mammalian Phenotype Ontology; **(B**–**D)** Overrepresented categories were found relating to ocular function (GO categories: ‘visual perception’, ‘response to light stimulus’, ‘sensory perception of light stimulus’, ‘retina development in camera-type eye’; Pathways: ‘the phototransduction cascade’ ‘inactivation, recovery and regulation of the phototransduction cascade’; Phenotype: electrophysiology, morphology, and degeneration of the retina, rods, and cones), various RNA processing, splicing, and metabolism functions (GO categories: ‘RNA processing’, ‘rRNA processing’, ‘mRNA processing’, ‘RNA splicing’, via transesterification reactions’, ‘mRNA splicing, via spliceosome’, ‘rRNA metabolic process’, ‘ncRNA metabolic process’, ‘mRNA metabolic process’, ‘RNA transport’), and direct responses to the physical pressures of spaceflight (GO categories: ‘response to abiotic stimulus’, ‘response to radiation’, ‘cellular response to stress’).

The space environment caused a wide array of changes in gene expression, the most prominent were changes in ocular pathways and RNA processing. Twenty mice spent 35 days on the International Space Station as part of NASA’s ninth Rodent Research experiment (RR-9). An additional twenty mice were housed on Earth in the same housing hardware used in flight for the ground control group. RNA sequencing was performed on sixteen mice (n = 8/group). 600 differentially expressed genes (DEGs) were identified between the spaceflight and ground control groups using DESeq2^11^ with an adjusted p-value threshold of 0.01 (Sup. Table 1). Hierarchical clustering was performed on the DEGs according to their expression values to determine which mice have similar DEG expression profiles. Mice clustered according to their inclusion in the ground control or spaceflight group. The spaceflight group had 286 upregulated genes and 314 downregulated genes compared to the ground control (Fig. 1A).

An overrepresentation analysis (ORA) of gene ontology (GO), pathway, and phenotypic categories was performed on the DEGs using WebGestalt^12^. From this analysis, 46 GO, 5 pathway, and 14 phenotypic categories were found with a false discovery rate (FDR) of less than 0.05 (Fig. 1B–D, Sup. Table 2). Included in the overrepresented categories are categories related to ocular function (GO categories: ‘visual perception’, ‘response to light stimulus’, ‘sensory perception of light stimulus’, ‘retina development in camera-type eye’; Pathways: ‘the phototransduction cascade’ ‘inactivation, recovery and regulation of the phototransduction cascade’; Phenotype: electrophysiology, morphology, and degeneration of the retina, rods, and cones), various RNA processing, splicing, and metabolism functions (GO categories: ‘RNA processing’, ‘rRNA processing’, ‘mRNA processing’, ‘RNA splicing’, via transesterification reactions’, ‘mRNA splicing, via spliceosome’, ‘rRNA metabolic process’, ‘ncRNA metabolic process’, ‘mRNA metabolic process’, ‘RNA transport’), and direct responses to the physical pressures of spaceflight (GO categories: ‘response to abiotic stimulus’, ‘response to radiation’, ‘cellular response to stress’).

Some genes were highly differentially expressed, but were not included in any of the GO category, pathway, or phenotype gene lists from the ORA (Sup. Fig. 1). *Drd4* was the most significantly differentially expressed gene and was upregulated in the spaceflight group (adjusted p-value: 4.31E-51; log2 fold-change: 0.812). *Drd4* is a dopamine receptor that control circadian rhythm in the mammalian retina^13^. Similarly, *Hist1h2bc* was not found in any categories of the ORA, but was upregulated in the spaceflight group (adjusted p-value: 1.66E-09; log2 fold-change: 0.526). *Hist1h2bc* has been previously shown to be upregulated in the aging retina^14^. Together, these genes support previous studies suggesting that spaceflight disrupts circadian rhythms and is a potential model for aging^15,16^.

### Genes associated with retinitis pigmentosa are differentially expressed in the space environment

We sought to determine whether any of the DEGs from the spaceflight samples were also differentially expressed in common retinal diseases. We compiled a list of disease-associated genes for the following retinal diseases: retinitis pigmentosa, diabetic retinopathy, age-related macular degeneration, and retinal detachment. Human disease-associated gene lists were found using the DisGeNET database^17^. Genes in the DisGeNET database were filtered by their gene-disease association score (GDA). Only genes with a GDA greater than 0.2 were included in the analysis. This included disease-associated genes from expertly curated and animal model databases, but excluded disease-associated genes from inferences and text-mining databases. Disease-associated genes were converted to their mouse ortholog using the Mouse Genome Informatics database^18^. Genes that did not pass the DESeq2 threshold for the number of mean counts (i.e., they had an adjusted p-value equal to ‘NA’), were filtered out from the analysis.

**Figure 2:**
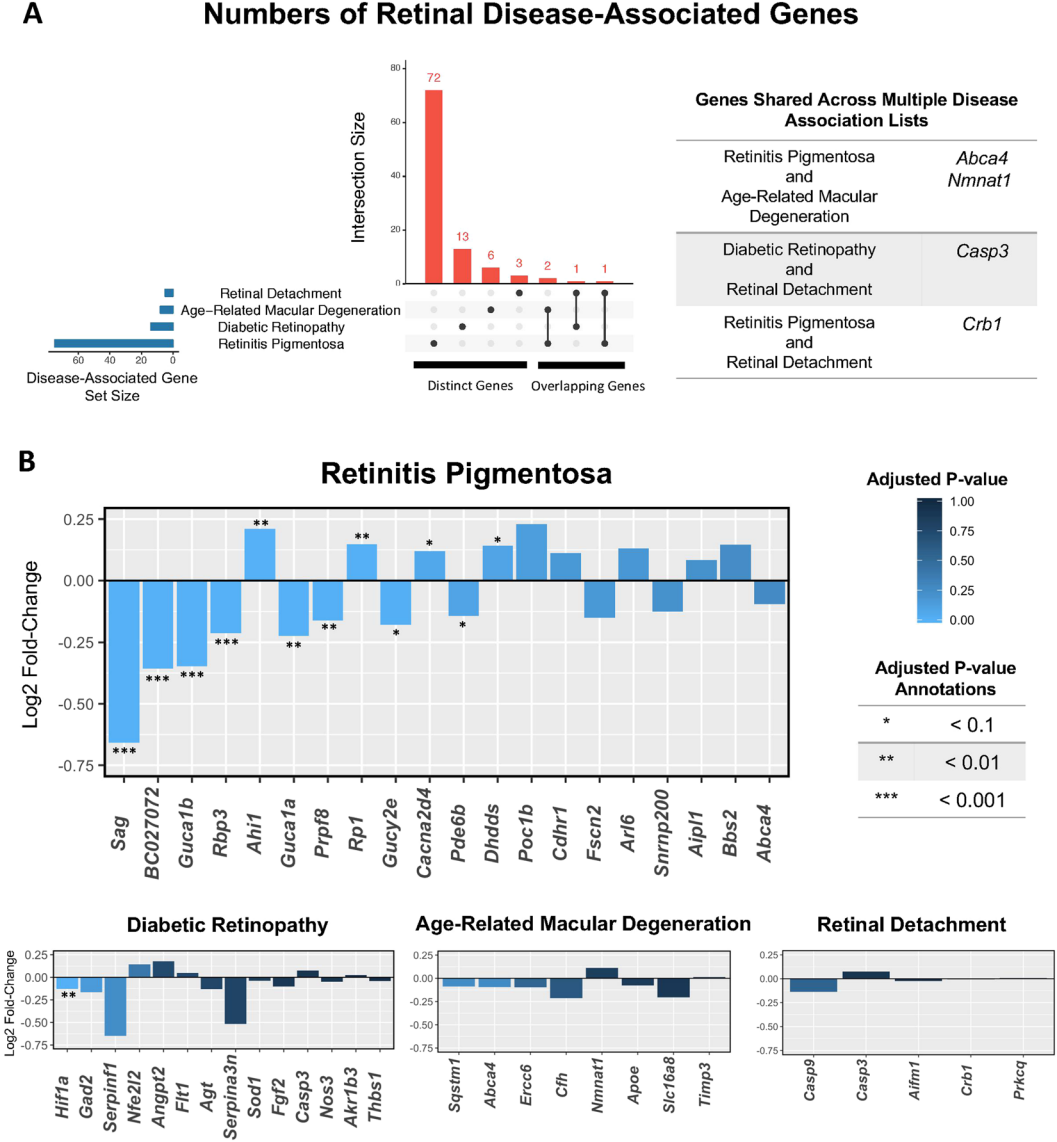
Retinal disease-associated gene expression. (**A**) UpSet diagram showing the number of genes in each disease set from DisGeNet. Genes that are distinct to each set are in the first four columns. Genes shared among sets are in the last three columns; **(B)** Log2 fold-change values of disease-associated genes. Genes are plotted from left to right in order of adjusted p-value. Bars are colored by the magnitude of the adjusted p-value. Adjusted p-values are further annotated based on their order of magnitude. The plot for retinitis pigmentosa displays the top 20 most significant genes based on adjusted p-value. All other diseases are displaying all of their associated genes.

Present in the DisGeNet database were 75 genes associated with the disease retinitis pigmentosa, 14 genes with diabetic retinopathy, 8 genes with age-related macular degeneration, and 5 genes with retinal detachment (Sup. Table 3). Most of the disease-associated genes were unique to a single retinal disease, with the exception of *Abca4*, *Nmnat1*, *Casp3*, and *Crb1*, which were each shared across two diseases (Fig. 2A). None of the disease-associated genes that were shared across multiple diseases were differentially expressed in spaceflight.

Of the 75 genes associated with retinitis pigmentosa, 12 were differentially expressed during spaceflight (*Sag, BC027072/Pcare, Guca1b, Rbp3, Ahi1, Guca1a, Prpf8, Rp1, Gucy2e, Cacna2d4, Pde6b, Dhdds*), the most of any of the four retinal diseases examined. Diabetic retinopathy had one disease-associated gene that was differentially expressed during spaceflight (*Hif1a*). Age-related macular degeneration and retinal detachment disease-associated genes were not differentially expressed during spaceflight (Fig. 2B).

### Differentially expressed transcription factors hint that spaceflight changes chromatin organization

**Figure 3:**
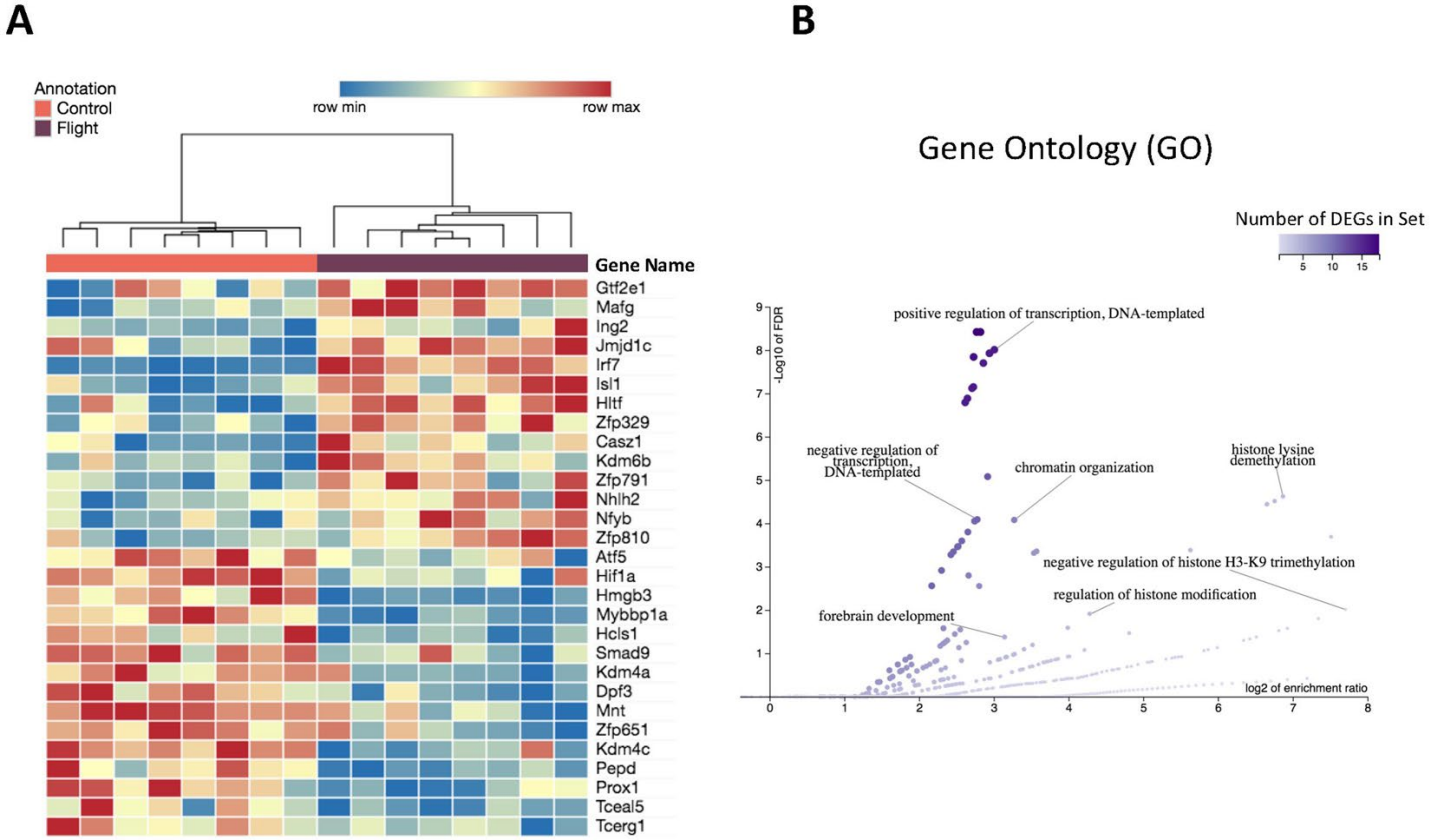
Transcription factor clustering and functions between spaceflight and control mice. (**A**) Hierarchical clustering of the 29 DETFs between spaceflight and ground control mice; **(B)** Enriched gene ontology (GO) biological process categories for DETFs.

DEGs reported in Fig. 1A were filtered by their PANTHER protein class in order to find differentially expressed transcription factors (DETFs)^19^. Out of the 600 DEGs, 29 are DETFs. Hierarchical clustering was performed on the DETFs according to their expression values. Mice clustered according to their inclusion in the ground control or spaceflight group (Fig. 3A). Eight of the DETFs are necessary for development and maintenance of the retina and other structures of the eye: *Mafg*, *Isl1*, *Atf5*, *Kdm6b*, *Hmgb3*, *Prox1*, *Casz1* and *Hif1a*^20–27^. Of special interest are *Cazs1* and *Hif1a*. *Cazs1* is a heterochromatin regulator that acts in a splice-variant specific manner^24^ and *Hif1a* inactivation has been shown to significantly mitigate photoreceptor degeneration in a chronic hypoxia-like stress model^25^.

An overrepresentation analysis (ORA) of gene ontology (GO) terms was performed on the DETFs. 47 GO categories were found with an FDR of less than 0.05 (Sup. Table 4). The affinity propagation filter from WebGestalt was applied in order to find a reduced number of GO terms that are representative of all GO enrichment results. We found enrichment of GO categories related to DNA transcription (‘positive regulation of transcription’, DNA-templated, ‘negative regulation of transcription, DNA-templated’), further affirming the role of these genes as transcription factors. Additionally, there were enriched GO categories relating to chromatin organization, including regulation of histone modification, histone lysine demethylation, negative regulation of histone H3K9 trimethylation (Fig. 3B). The genes in these GO categories include *Kdm4a, Kdm4b, and Kdm6b*, which are known regulators of lysine methylation. *Kdm4a* and *Kdm4b* are regulators of H3K9me2/me3^28^, which is a regulator of constitutive heterochromatin and an indicator for the presence of constitutive heterochromatin. *Kdm6b* is a regulator of H3K27me2/me3^28^ and is primarily responsible for the silencing of gene expression.

### The space environment decreases the thickness of retinal tissue and increases oxidative stress and cone photoreceptor damage

**Figure 4:**
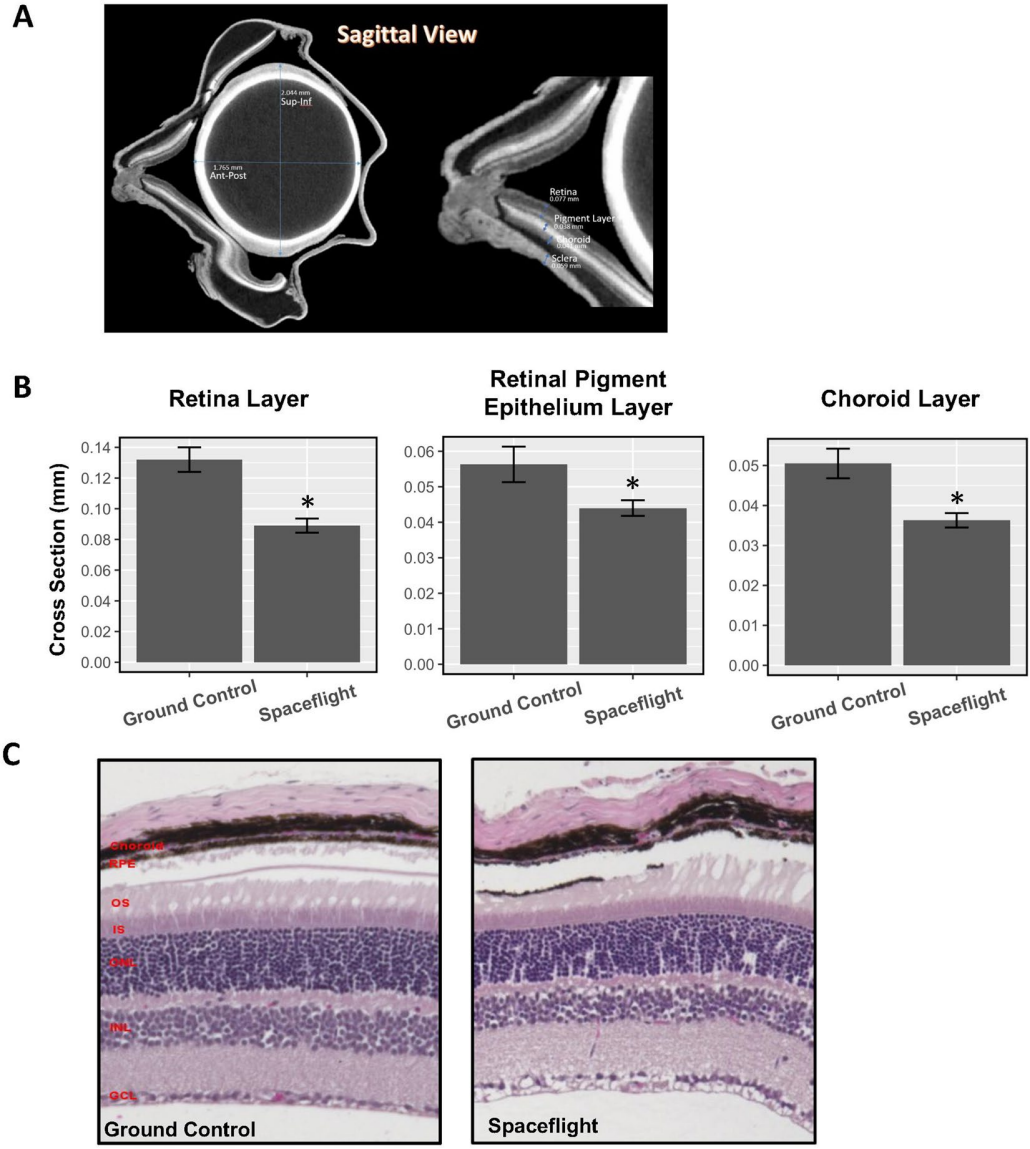
Spaceflight decreases the thickness of multiple layers of the eye. (**A**) Sagittal view of a ground control mouse. Layers of the eye on the right side of the image are annotated, from top-to-bottom, retina (0.077 mm), retina pigment layer (RPE, 0.038 mm), choroid (0.041 mm), sclera (0.059 mm); **(B)** Average thickness of the retinal layer, RPE layer, and the choroid layer measured by MicroCT in the spaceflight and control groups. Counts were averaged across five retinas per group. Values were represented as mean thickness + standard error (SEM). SEM of the mean is marked with error bars. Significantly lower in cross section thickness in the spaceflight group compared to the ground control group is denoted ‘*’ (p < 0.05). **(C)** Cross sections of the retina from control and spaceflight mice. GCL: ganglion cell layer; INL: inner nuclear layer; ONL: outer nuclear layer; IS: inner segment; OS: outer segment, RPE: pigment epithelium layer.

Micro-computed tomography (MicroCT) images were generated in order to characterize global ocular morphology and to measure the thickness of the retina and surrounding tissues for ground control and spaceflight mice (Fig. 4A). Total retina, retinal pigment epithelium (RPE), and choroid layers of the eye decreased significantly in thickness (p < 0.05) during spaceflight (Fig. 4B). Additionally, choroid deformation and folds were noticed in spaceflight mice (Fig. 4C).

**Figure 5:**
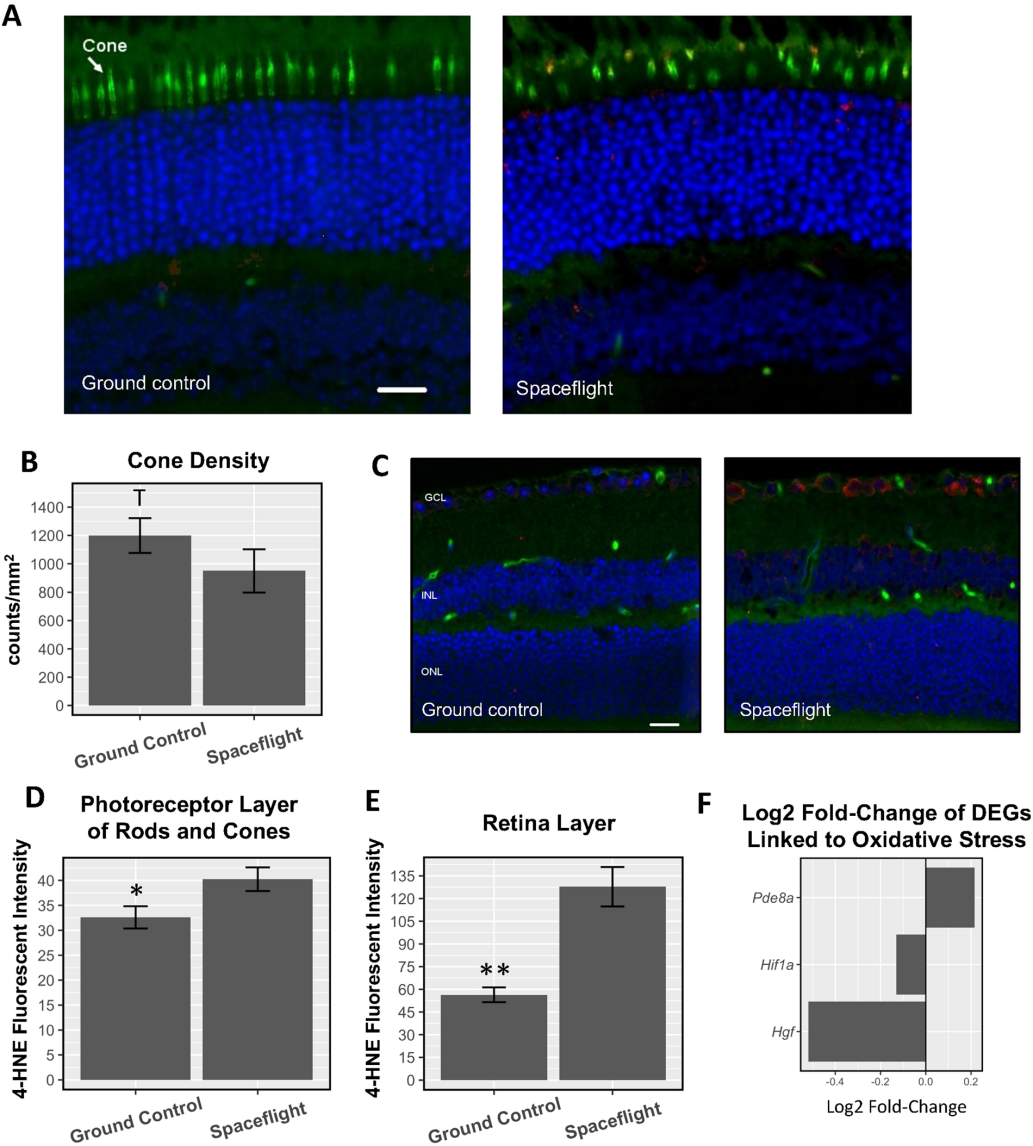
Spaceflight causes photoreceptor degradation and oxidative stress. (**A)** Immunofluorescence staining for PNA, a marker for cone photoreceptors (green), and HNE, a marker for oxidative stress (red), in the photoreceptor layer. The nuclei were counterstained with DAPI (blue); Scale bar = 50 mm. **(B)** Cell density for PNA–positive cone photoreceptors; (**C**) Immunofluorescence staining for HNE (red) in the retina layer. Scale bar = 50 µm; **(D)** Fluorescent intensity of the HNE marker in the photoreceptor layer of rods and cones (arrow); **(E)** Fluorescent intensity of the HNE marker across the retina; **(F)** Log2 fold-change of DEGs under the GO category “negative regulation of oxidative stress-induced cell death”; (**B**,**D**,**E**) Counts were averaged across five retinas per group. Values are represented as mean density ± SEM. Significance values of the spaceflight group compared to ground controls are denoted with ‘*’ (p < 0.05), ‘**’ (p < 0.01), and ‘T’ (strong trend differences between spaceflight and ground controls; p = 0.06).

Immunofluorescence staining with peanut agglutinin (PNA), a specific marker for cone photoreceptors, was performed in order to view cone photoreceptors in the eye. Cone photoreceptors show signs of degradation during spaceflight (Fig. 5A). Our quantitative assessment (Fig. 5B) revealed a strong trend decrease in density of cones in the spaceflight mice (950 counts/mm^2^) compared to ground control group that had an average of 1199 counts/mm^2^ (p = 0.06).

The occurrence of lipid peroxidation was evaluated with antibody against 4-hydroxynonenal (4-HNE), which is an indicative marker of oxidative damage to the retina. Increased 4-HNE staining was seen in cone photoreceptors, retinal inner nuclear layer (INL), and ganglion cell layer (GCL) after spaceflight compared to ground controls (Fig. 5C). As shown in Fig. 5D, the fluorescent intensity profiles in the photoreceptor cones which reflects endogenous level of HNE, was also significantly increased (p < 0.05) in the spaceflight group compared to the ground controls. Fluorescent intensity from the HNE marker was also significantly increased in the GCL/INL of the spaceflight group for the retina (p < 0.01) (Fig. 5E). Our RNA-seq experiment identified three DEGs - *Pde8a, Hif1a, Hgf* - that are part of the GO Biological Process category for ‘negative regulation of oxidative stress-induced cell death’ (GO:1903202) (Fig. 5F). *Pde8a* is a member of the phosphodiesterase family. Phosphodiesterase specific member inhibition has been previously shown to attenuates oxidative stress in diverse tissues^29–32^. *Hif1a* was shown to protect against oxidative stress directly at the mitochondria^33^, but the effects of this gene were shown to be highly contextual in the presence of reactive oxygen species^34^. *Hgf* activates an antioxidant signaling pathway that culminates in increased levels of nitric oxide and the activity of antioxidant enzymes regulated by this molecule^35,36^. Taken together, the upregulation of *Pde8a* and the downregulation of the *Hif1a* and *Hgf* are linked to the development of increased oxidative stress in the retina.

## Discussion

We have found a distinct gene expression signature in the retina of mice exposed to the spaceflight environment compared with the ground control. This signature is enriched for genes related to visual perception, the phototransduction pathway, and numerous retina and photoreceptor phenotype categories. Other studies have also shown changes of gene expression involved in cell structure, growth, migration and adhesion in the retinal cells exposed to simulated microgravity^37,38^. Less anticipated was the overrepresentation of genes related to RNA processing, metabolism, and transport pathways for the first time described as relevant for spaceflight in mammals. A recent study in *Arabidopsis thaliana* has discovered mRNA isoforms created by alternative splicing that are unique to their spaceflight exposed samples^39^. Our results indicate that in addition to *Arabidopsis thaliana*, there may be splice isoform alterations in mammals during spaceflight as well that should be explored in future work and included in the risk assessment for long duration space missions.

Differential RNA splicing has also been implicated as a potential disease-causing mechanism for retinitis pigmentosa. Mutations in the gene *Rho*, which creates a protein that composes rod photoreceptors and is essential for vision in low-light conditions, can alter its splice isoforms. These alternate isoforms lead to the construction of abnormal RHO proteins and trigger the pathology of retinitis pigmentosa^40^. Additionally, proper alternative splicing of *Nxnl1* from rod photoreceptors and other ocular cell types has been associated with cone photoreceptor maintenance^41,42^. Disruption of these splice isoforms can reduce the density of cone photoreceptors and the thickness of the outer nuclear layer of the retina^43^. Overall, our results indicate that the space environment might create unique epigenetic events and differential RNA splicing, in addition to the altered gene expression profile reported. Future studies should address which genes are alternatively spliced to understand potential changes in protein structure and find further associations with retinal diseases. Furthermore, mouse models for retinitis pigmentosa are available. Determining which mouse model is most similar to mice exposed to the space environment will help determine the appropriate model to perform Earth-analog experiments. Finding an appropriate Earth-based analog will accelerate the speed of research dedicated to finding a countermeasure for the retinal damage caused by spaceflight.

Several transcription factors were identified as necessary for development and maintenance of the retina and other structures of the eye^20^. Although eight of the DETFs are known for their importance for the retinal development and maintenance in Earth conditions, 21 of the DETFs have no reports in the literature linking them to the eye tissue. From the DETF overrepresentation analysis, transcription factors were identified that are known to influence chromatin architecture. Changes in chromatin architecture indicate that the cause for changes in retinal structure observed during spaceflight is more complex than solely alterations in RNA processing. The overrepresentation analysis showed enrichment of transcriptional regulators for H3K9 demethylation, which is a marker for constitutive heterochromatin. Demethylation of H3K9 can result in the unraveling of this heterochromatin, which can then influence the accessibility of genes and regulatory elements in the genome^44^. This can further drive changes in gene expression and either create abnormal and potentially harmful cellular behavior or act as a compensatory mechanism to mitigate damage the retinal tissue has experienced from spaceflight. The connections between chromatin reorganization as a disease-causing or disease-mitigating mechanism during spaceflight has not yet been explored. Determining the locations of chromatin reorganization could provide insight to these questions. Furthermore, the literature does not report any of the DETFs directly related to retinitis pigmentosa, which could indicate that these genes, although not yet described, could have a role in retinitis pigmentosa or that they are part of spaceflight specific expression network that can be explored as biomarkers and/or drug targets.

Compensatory mechanisms to mitigate cellular damage have been observed in response to oxidative stress. Our immunostaining for marker 4-HNE showed evidence of oxidative stress within the retina. Evidence of a response to this oxidative stress was also observed in the gene expression data. The gene *Bnip3* was differentially expressed, showing upregulation during spaceflight (adjusted p-value: 2.94E-03; log2 fold-change: 0.309). *Bnip3* is known for its role in removing misfolded proteins from the cell^45^. Misfolded proteins can occur as a result of oxidative stress. Also upregulated in the spaceflight data are heat shock genes *Hsp90aa1*, *Hsp90b1*, and *Hspa4l* which can assist correct protein folding and are known to protect against oxidative stress^46,47^. From this, we see evidence of a potential compensatory mechanism becoming activated in spaceflight in order to correct and mitigate cellular damage accumulating in retinal tissue. One consideration for long duration space missions are the compensatory mechanisms that are activated in the space environment. For long duration missions, it becomes imperative to evaluate for how long these compensatory mechanisms can be sustained and how to extend or amplify its beneficial effects. Additionally, the nearest Earth-analog disease that we identified, retinitis pigmentosa, has known links to oxidative stress and mitochondrial dysfunction. Although no cure is currently known, supplementation with certain minerals and vitamins, especially vitamin A, has been demonstrated to delay the disease progression^48^ and should be considered a possible countermeasure to spaceflight induced retinal damage.

We also found evidence that the circadian rhythm of the retina was altered in spaceflight. The circadian rhythm regulates behavioral and physiological processes over a 24-hour period, principally controlled by day/night patterns. Disruptions in the circadian rhythm can lead to irregular sleep patterns, mood, and metabolism function, as well as the development of cancer^49^. The differentially expressed gene with the smallest adjusted p-value, *Drd4* (adjusted p-value: 4.31E-51; log2 fold-change: 0.812), is a dopamine receptor that has been associated with the circadian rhythm. Specifically, *Drd4* downregulates the gene *Adcy1*^13^, which was also found to be differentially expressed in this study (adjusted p-value: 1.45E-09; log2 fold-change: −0.427). *Adcy1* produces cyclic AMP, a signalling mechanism in numerous retinal functions, such as retinomotor movements, disc shedding, certain types of retinal degeneration, and apoptosis of photoreceptors^50–52^. Circadian rhythm genes are of special interest in the space environment, where day/night light signals are irregular. For example, astronauts aboard the ISS will experience up to 16 sunsets and sunrises a day^53^. However, the mice in our experiment were in constant 12-h light/dark cycle, which leads us to believe that events other than the light/dark cycle itself are driving the changes in the circadian rhythm.

Increased photoreceptor cone damage, reduced thickness of the retinal ONL, retinal INL, RPE, and choroid layers of the eye in the spaceflight group compared to ground control were reported in our study. Proton radiation-induced damage to the photoreceptor layer was also observed in our previous rodent study^54^. It is important to note that changes in global ocular structure and retinal layer thickness could have clinical and/or pathological significance^34^. The ONL contains the nuclei of the photoreceptor cells and the INL contains the cell bodies of bipolar, horizontal, amacrine, and Müller cells. Our published study from a Space Shuttle Mission (STS-135) showed a significant increase of apoptosis in the ONL and INL of the photoreceptor layer in spaceflight mice^2^. Photoreceptor loss is responsible for irreversible blindness in many retinal diseases^55^ and photoreceptors are more sensitive to environmental damage^56^. Retinal damage, retinal photoreceptor cell loss, and thinning of the inner retina precede subsequent capillary degeneration^57,58^. Our findings strongly implicate the space environment causes retinal injury and the potential development and progression of retinal degeneration.

The potential benefit of micro-CT in quantitative evaluation of ophthalmologic structure was encouraging. Our results showed degradation in ocular structures by spaceflight, particularly of the retina, RPE, and choroid layers of the eye, as evidenced by their decreased thickness. MicroCT provides an easy and non-destructive technique to image without any modification. Additionally, it shows the entire region of interest digitally, thereby increasing accessibility and reproducibility of the findings. Future work should use volumetric data to perform other analyses that take advantage of the micro-CT imaging capabilities, since it has been used successfully for studying many normal and pathologic tissues^59^.

One area of concern is whether observed degradation of photoreceptors, retinal damage, and ocular structure alteration will persist or whether it will reverse once the organism is returned to Earth. Future studies should investigate the long-term impact of observed structural changes of the retina and retinal photoreceptors function that may lead to late retinal degeneration. Additional studies are needed to determine which aspects of spaceflight are responsible for photoreceptor damage and oxidative stress. Leading causes include radiation and possible alterations in intracranial pressure (ICP) or intraocular pressure (IOP)^60,61^ induced by microgravity. It is possible that both space radiation and microgravity work in conjunction with one another to cause a more severe pathological result than either would in isolation. Other factors that may be driving differential gene expression include increased stress an organism experiences due to the physiological pressures of spacecraft takeoff and landing and the psychological pressures of spaceflight. The body size of an organism also impacts the amount of interstitial fluid per gram of body weight, which could impact the magnitude of fluctuating pressures influencing the retina^62^. Studies using larger organisms could better model the degree of fluctuating pressures experienced by humans during spaceflight.

There are many potential avenues left to explore in order to understand the response of the mammalian retina to spaceflight. In this study we have characterized the physical response of the retina, the degradation of photoreceptors, and the presence of oxidative stress markers. We have also observed several genes with significant differential expression in the spaceflight condition were also differentially expressed in the disease retinitis pigmentosa. Additionally, we suspect the changes that we are observing during spaceflight are influenced by alternative splicing and chromatin reorganization. There remains much work to be done on understanding the mechanism of spaceflight-induced oxidative stress on retinal damage. Additional work will need to be done to separate disease-causing changes in gene expression from compensatory changes. We also believe these data will be useful for understanding other spaceflight-related health concerns, such as misregulation of the circadian rhythm and accelerated aging. A full list of DEGs has been provided as a resource (Sup. Table 1) toward further understanding of associations between spaceflight, retinal function, and overall mammalian health.

## Methods

### Flight and control conditions

SpaceX successfully launched the 12th Commercial Resupply Service (CRS-12) payload at the Kennedy Space Center (KSC) on a 35-day mission in August, 2017. 10-week-old male C57BL/6 mice (n = 20) (Jackson laboratories, Inc. Bar harbor, ME) were flown for NASA’s ninth Rodent Research experiment (RR-9). The mice lived in NASA’s Rodent Habitats (RH) aboard ISS for 35 days before returning to Earth via SpaceX’s Dragon capsule. All mice were maintained at an ambient temperature of 26–28 °C with a 12-h light/dark cycle during the flight. GC mice were placed into the same housing hardware used in flight, and environmental parameters such as temperature and carbon dioxide (CO_2_) levels were matched as closely as possible based on telemetry data. GC mice were fed the same special NASA food bar diet as the space flown mice. All mice received the same *ad libitum* access to food and water. The study followed the recommendations in the Guide for the Care and Use of Laboratory Animals of the National Institutes of Health (NIH) and was approved by the Institutional Animal Care and Use Committee (IACUC) of Loma Linda University (LLU) and The National Aeronautics and Space Administration (NASA).

### Post-flight evaluation of the mice

Upon return to the Earth, mice were transported to Loma Linda University (LLU) within 28 hours of splashdown. At LLU, animals were removed from the animal enclosure hardware and assessed for survival and health. It was reported that all the mice survived the 35-day space mission and were in good condition, i.e. no obvious deficiencies/abnormalities as described by the inspecting personnel.

### Dissecting and preservation of mouse eyes after spaceflight

The mice were rapidly euthanized in 100% CO_2_ and eyes were collected within 38+/−4 hours of splashdown (n = 20/group). The retinas from rights were dissected and placed individually in sterile cryovials, snap frozen in liquid nitrogen and kept at −80 °C prior to use. The left eyes were fixed in 4% paraformaldehyde in phosphate buffered saline (PBS) for 24 hours and then rinsed with PBS for immunohistochemistry (IHC) assays. The right eyes from 2 experimental groups: ground control and spaceflight (n = 8 mice per group) were dissected to obtain the retina. The retinas were flash-frozen and stored at −80 °C for genomic analysis.

### Immunostaining assays and histology

Paraffin-embedded sections (Six µm) of left eye, roughly 100 mm apart, were used for analysis (n = 6/group). To evaluate oxidative damage in the retina, Immunostaining was performed on ocular sections using 4-HNE antibody specific for lipid peroxidation. Sections were incubated with the anti-4-HNE antibody (catalog no. HNE11-S, Alpha Diagnostic International Inc., San Antonio, TX) at 4 °C for 2 hours followed by a donkey anti-rabbit IgG fluorescence-conjugated secondary antibody (catalog no. A21206, Invitrogen Corp., Waltham, MA) for 2 hours at room temperature and counterstained with DAPI. For double labeling of HNE and PNA, sections were incubated with fluorescein isothiocyanate (FITC)-conjugated Peanut agglutinin (1:100 in 1% BSA) for an hour at room temperature for labeling the photoreceptor cones. Sections were then incubated overnight (18-21 hours) at 4 °C with primary antibody against rabbit anti-4-HNE (Alpha Diagnostic Intl. Inc. San Antonio, TX). After washing three times in PBS, sections were further treated with secondary antibody Alexa Fluor 568 goat anti-rabbit IgG (Life Technologies, Eugene, Oregon). PNA was obtained from (Vector Laboratories, Burlingame, CA). The cell nuclei were counterstained with DAPI and were mounted and coverslipped with Vectashield Hard-Set Mounting Medium (Vector Laboratories). To characterize layers of retinal structure, a series of 6 µm sections were also stained with hematoxylin and eosin (H&E). Images were captured with a BZ-X710 All-in-One inverted fluorescence microscope. In each sample, a total of 5 fields of images were sampled systematically at 20X magnification spanning the entire retina sections.

### Quantification of immunostaining

For quantitative analysis, the number of PNA- positive cells were counted in three sections of tissue from each animal using Image J counting plugin 1.41 software (National Institutes of Health, Bethesda, MD; http://rsbweb.nih.gov/ij/). The density profiles were expressed as mean number of PNA cells/mm^2^, and counts were averaged across each group. To determine HNE immunoreactivity, fluorescence intensity was measured on 5 randomly selected fields on each section and calculated using Image J software. The data were extracted and averaged within the group. A detailed method was described in Mao *et al*.^2^.

### Sample preparation for MicroCT scanning

A subset of eye samples (n = 6/group) were fixed in 4% formaldehyde in PBS. After fixation, the mice eyes were dehydrated in ethanol. To prevent an abrupt shrinkage of the fixed sample, a graded series of ethanoic solutions were used. Afterwards, the mice eyes were stained in 10% phosphomolybdic acid solution (PMA) for 6 days. Samples were washed in absolute ethanol and then placed each eye in the same rotation in individual 2 mL plastic containers filled with absolute ethanol for scanning.

### Micro CT scanning and analysis

Samples were scanned using a high resolution micro-computed tomography system (SkyScan 1272 desktop micro-CT system, Bruker, Kontich, Belgium), with an accelerating source voltage of 50 keV, a source current of 80 mA with an integration time of 90 min. During the scanning process, the samples were rotated at 180 degrees, with an imaging voxel size of 4.5 um, frame averaging of 4, rotation step of 0.4 and no filter. These images were saved for the reconstruction of the 3D object. The thickness of the retina and surrounding tissues were measured on MicroCT images by descriptive analysis. Measurements of the lens, retina, RPE layer, choroid and sclera were performed in the middle slice of the sagittal view of each sample using the optical nerve as reference. Three measurements were recorded of each structure to finally obtain an average.

### RNA/DNA extraction

Retina tissues (n = 8/group) were homogenized with Precellys CKM beads (Hilden, Germany) in 600 µl of RLT plus buffer. Total RNA and DNA were extracted using the QIAGEN AllPrep DNA/RNA/miRNA Universal Kit according to the manufacturer’s protocol. After isolation, RNA and DNA were frozen and kept at −80 °C until further use. Isolated RNA samples were quantified and purity checked by NanoDrop spectrophotometry (Thermo Fisher Scientific, Chino, CA). DNA and RNA were further quantified using the Qubit dsDNA High Sensitivity Kit and RNA Broad Range Kit, respectively (Life Technologies, Carlsbad, CA). RNA quality was evaluated using the Agilent 2200 TapeStation and RNA ScreenTape with 1ul of randomly selected RNA denatured at 72 °C for three minutes per manufacturer’s instructions (Santa Clara, CA). RNA integrity numbers (RIN) of tested RNA samples had values between 7.7 and 8.9.

### RNA-Seq library construction

The Ovation® Mouse RNA-Seq System 1-16 (NuGEN Technologies, #0348) was used per manufacturer’s instructions to construct stranded RNA-seq libraries. 100 ng of total RNA was used as input. First and second strands of cDNA were synthesized from total RNA (100 ng) spiked with 1 µl of 1:500 diluted ERCC ExFold RNA Spike-In Mix 2 (Life Technologies, Carlsbad, CA) at the appropriate ratio. Following primer annealing and cDNA synthesis, the products were sheared using Covaris S220 Focused-ultrasonicator (Covaris Inc., Woburn, MA). 130 µl of each sample was sheared according to manufacturer’s instructions. The parameters were set as follows: 10% duty factor, peak power 175 and 200 cycles per burst at 4 °C for 200 seconds to obtain fragment sizes between 150–200 bp. This was followed by end-repair, adaptor index ligation and strand selection. Strand selection was performed by using custom InDA-C primer mixture SS5 Version5 for mice with cytoplasmic and mitochondrial ribosomal RNA depletion. Finally, libraries were amplified using 17 cycles (Mastercycler^®^ pro, Eppendorf, Hamburg, Germany), and purified with RNAClean XP Agencourt beads.

### Library quantification and quality control (QC) test

The final amplified libraries were purified using Agencourt RNAClean XP beads (Beckman Coulter, Indianapolis, IN) and quantified using Qubit dsDNA HS Kit on Qubit 3.0 Fluorometer (Life Technologies, Carlsbad, CA). Quality and peak size was determined using D1000 ScreenTape on the Agilent 2200 TapeStation (Agilent Technologies, Santa Clara, CA).

### Sequencing

Final libraries were each diluted to 4 nM and further quantitated to ensure high accuracy quantification for consistent pooling of barcoded libraries and maximization of the number of clusters in the Illumina flow cell. Libraries of different indices were pooled for sequencing together in equimolar amounts. Pooled libraries were quantified by Qubit prior to sequencing. For sequencing, clusters were generated on cBot with HiSeq3000/4000 PE cluster kit (Illumina, Inc., San Diego, CA) from 5 ul of 2 nM final library pool (200 pM at cBot loading). Sequencing was performed on an Illumina HiSeq4000 (Loma Linda University Center for Genomics) using 75 cycles SBS reagents (Illumina, Inc., San Diego, CA). Single-indexed and single-end reads with 75 nucleotides length (1 × 75 bp) were generated. Fastq files generated from the sequence run were demultiplexed (Loma Linda University Center for Genomics), quality checked (quality score >38), trimmed from adapters with Trimmomatic (version 0.01) and mapped against mouse reference genome (NCBI_build_37.2) with TOPHAT2 (version 2). Overall mapping rate was ~85%. Expressed transcripts were counted with Multi HTSeq (version 0.02).

### Computational pipeline/RNA-Seq analysis tools

Differential Expression analysis was carried out using raw counts from input to the R package DEseq2 Version 1.22.2 using R version 3.5.1. A gene was considered differentially expressed (DE) if was below a false discovery rate (FDR) adjusted p-value of 0.1^63^. Heatmaps were generated using the webtool Morpheus, from the Broad Institute (https://software.broadinstitute.org/morpheus/). Hierarchical clustering was performed on rows and columns using one minus Pearson correlation. DEGs that are transcription factors were annotated using the Panther database version 14.1^19^.

GO, gene network analysis, and phenotype graphs were created by performing an overrepresentation enrichment analysis using WebGestalt^12^. GO analysis was carried out using biological process database. Gene network analysis was carried out using the Reactome database^64^. Phenotype analysis was carried out using mammalian phenotype ontology. FDRs were calculated using the Benjamini-Hochberg procedure.

Human disease associated genes were found for the diseases retinitis pigmentosa, age-related macular degeneration, diabetic retinopathy, and retinal detachment using the database DisGeNET version 6.0 with a gene-disease association (GDA) score greater than 0.2^17^. Human genes were converted to their mouse orthologs using the Mouse Genome Informatics database^18^. The genes KIZ, PCARE, RP2, and RP9 did not have their orthologs listed in the Mouse Genome Informatics database and were manually annotated using the GeneCards database (v4.10.0 Build 4)^65^.

## Supplementary information

Supplementary Figure 1

DEGs and Protein Classes

WebGestalt Results, DEGs

Gene-Disease Associations

WebGestalt Results, DETFs

## Data Availability

The datasets generated during and/or analysed during the current study are available in the GEO repository, under the accession identifier GSE131954.
