# Supplementary material for: Spaceflight influences gene expression, photoreceptor integrity, and oxidative stress-related damage in the murine retina
Source: Sci Rep. 2019 Sep 16;9:13304. doi: 10.1038/s41598-019-49453-x (PMC6746706; doi:10.1038/s41598-019-49453-x)
Supplement: Supplementary file 1 — Supplementary Figure 1 [file 41598_2019_49453_MOESM1_ESM.pdf]

Spaceflight influences gene expression, photoreceptor integrity, and oxidative stress-related damage in the murine retina.

Eliah G. Overbey[1] (*), Willian Abraham da Silveira[2], Seta Stanbouly[3], Nina C. Nishiyama[3], Gina RoqueTorres[4], Michael J. Pecaut[3], David Zawieja[5], Charles Wang[6], Jeffrey Willey[7], Michael Delp[8], Gary Hardiman[2], Xiao Wen Mao[3]

1 - University of Washington, Department of Genome Sciences, Seattle, WA, USA
2 - Queen's University Belfast, Faculty of Medicine, Health and Life Sciences, School of Biological Sciences, Institute for Global Food Security (IGFS),19 Chlorine Gardens, Belfast. Northern Ireland. BT9 5DL.
3 - Department of Basic Sciences, Division of Biomedical Engineering Sciences (BMES), Loma Linda University, Loma Linda, CA, 92350, U.S.A.
4 - Center of Dental Research, Loma Linda University, CA, 92354, U.S.A.
5 - Department of Medical Physiology, Texas A&M University, College Station, Texas, U.S.A
6 - Center for Genomics, Department of Basic Sciences, Loma Linda University, Loma Linda, CA, 92350, U.S.A
7 - Department of Radiation Oncology, Wake Forest School of Medicine. Bowman Gray Center, Winston-Salem, NC 27101, USA.
8 - Department of Nutrition, Food and Exercise Sciences, Florida State University, Tallahassee, FL, 32306, USA.

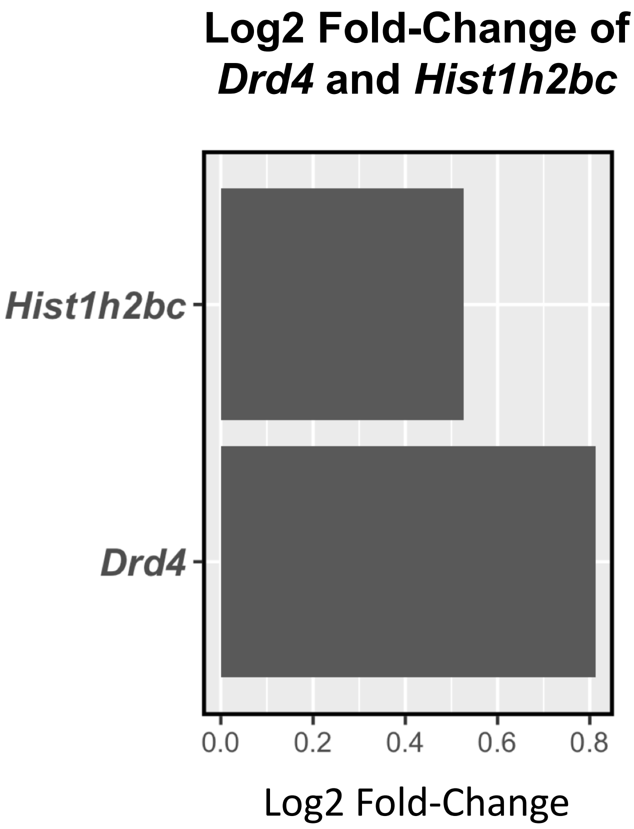

Supplementary Figure 1. Log2 fold-change of *Drd4* and *Hist1h2bc*. *Drd4* has an FDR of 4.31E-51 and is related to circadian rhythms. *Hist1h2bc* has an FDR of 1.66E-09 and is related to the aging retina.
